# Alterations in Immune Cell Profiles in the Liver in Diabetes Mellitus: A Systematic Review

**DOI:** 10.3390/ijms26094027

**Published:** 2025-04-24

**Authors:** Wanying Du, Elisha Siwan, Stephen M. Twigg, Danqing Min

**Affiliations:** 1 Greg Brown Diabetes and Endocrine Research Laboratory, Sydney Medical School (Central), Faculty of Medicine and Health, Charles Perkins Centre, The University of Sydney, Sydney, NSW 2006, Australia; wadu3218@uni.sydney.edu.au (W.D.); elisha.siwan@sydney.edu.au (E.S.); stephen.twigg@sydney.edu.au (S.M.T.); 2 Department of Endocrinology, Royal Prince Alfred Hospital, Sydney, NSW 2050, Australia

**Keywords:** diabetes, liver fibrosis, MAFLD, MASLD, MASH, NAFLD, NASH, immune cells, cytokines, inflammation

## Abstract

The aim of this study was to systematically review literature on immune responses in liver tissue pathology in diabetes, focusing on immune cell populations and related cytokines. A systematic search of relevant English full-text articles up to June 2024 from online databases, covering animal and human studies, was conducted using the PRISMA workflow. Thirteen studies met criteria. Immune cells in the liver, including monocytes/macrophages, neutrophils, and iNKT and T cells, were implicated in liver inflammation and fibrosis in diabetes. Pro-inflammatory cytokines, including interferon-ɣ, tumor necrosis factor-α, interleukin (IL)-15, IL-18, and IL-1β were upregulated in the liver, potentially contributing to liver inflammation and fibrosis progression. In contrast, the anti-inflammatory cytokine IL-4 was downregulated, possibly attributing to chronic inflammation in diabetes. Pathological immune responses via the TLR4/MyD88/NF-κB pathway and the IL-17/IL-23 axis were also linked to liver fibrosis in diabetes. In conclusion, this review highlights the putative pivotal role of immune cells in diabetes-related liver fibrosis progression through their regulation of cytokines and signaling pathways. Further research on diabetes and dysmetabolic liver pathology is needed to clarify immune cell localization in the liver and their interactions with resident cells promoting fibrosis. Targeting immune mechanisms may provide therapeutic strategies for managing liver fibrosis in diabetes.

## 1. Introduction

Diabetes and liver fibrosis are interrelated chronic metabolic diseases [1]. Around 70% of people with diabetes develop hepatic complications, including metabolic dysfunction-associated fatty liver disease (MAFLD) or metabolic dysfunction-associated steatotic liver disease (MASLD), formerly non-alcoholic fatty liver disease (NAFLD), and its severe form, metabolic dysfunction-associated steatohepatitis (MASH), or non-alcoholic steatohepatitis (NASH) [1]. In 2023, MAFLD/MASLD was officially defined through a multi-society Delphi statement to replace NAFLD, emphasizing a shift from focusing solely on the absence of alcohol consumption to recognizing its strong association with metabolic dysfunction, including obesity, type 2 diabetes mellitus (T2DM), insulin resistance, and other related conditions, while also replacing NASH with MASH to reflect disease progression [2]. In this review, MAFLD and NAFLD, as well as MASH and NASH, are used interchangeably, as the terminology prior to 2023 predominantly used NAFLD and NASH. NAFLD is the most common chronic liver disease, ranging from simple steatosis to NASH, which increases the risk of fibrosis, cirrhosis, and hepatocellular carcinoma [1]. Diabetes, particularly T2DM, is a major independent risk factor for NAFLD [1]. NAFLD affects 20–30% of those with type 1 diabetes (T1DM), and 65–80% with T2DM, 31% developing NASH and 15–25% with T2DM having severe liver fibrosis [3]. About 46% of those with T2DM and simple steatosis develop NASH over time [3]. Moreover, mortality is higher in those with both diabetes and chronic liver disease than in those with one or neither condition [4]. Despite the rise of MAFLD/NAFLD and MASH/NASH in diabetes, relatively little is known about the mechanisms and immune responses involved in such liver pathology.

**Figure 1:**
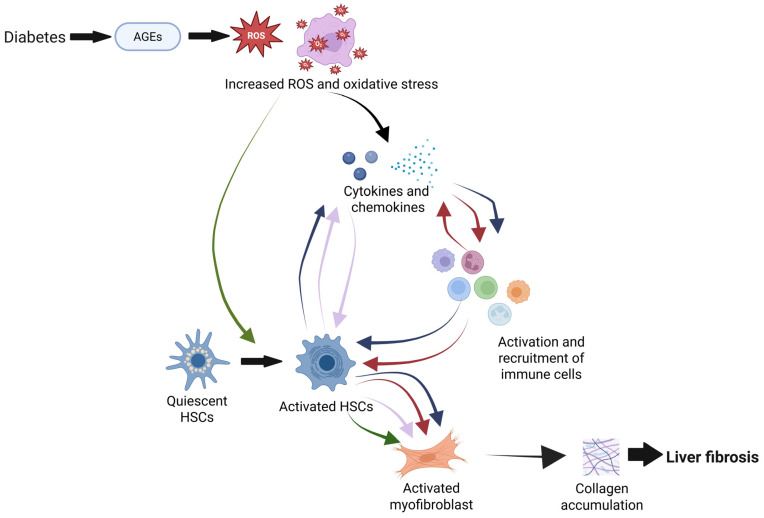
A current overview of the proposed pathogenic mechanism of diabetes-related liver fibrosis through the dysregulation of immune responses and activation of hepatic stellate cells. Diabetes promotes liver fibrosis through pathways including advanced glycation end products (AGEs)-induced oxidative stress, which, for example, affects hepatocytes in liver lobules. This stress triggers the release of cytokines and chemokines, leading to the activation of hepatic stellate cells (HSCs) and the recruitment of both specific and non-specific immune cells [6]. Immune responses regulate activated HSCs, the latter of which differentiate into collagen-producing myofibroblasts, resulting in collagen accumulation and fibrosis progression. Additionally, activated HSCs release cytokines that further stimulate immune cell activation, creating a reinforcing feed-forward loop that sustains HSC activation and amplifies the fibrotic response [6]. Color-coded arrows represent interactions between cytokines and chemokines (blue), immune cells (red), activated HSCs (purple), and activated myofibroblasts (green). Created with BioRender.com.

T2DM is characterized by insulin resistance and inadequate beta cell response, leading to hyperglycemia [5]. Chronic hyperglycemia raises advanced glycation end products (AGEs), leading to increased reactive oxygen species (ROS) and oxidative stress [6] (Figure 1). This activates transcription factors like activator protein-1 (AP-1) and nuclear factor-kappa B (NF-κB), promoting cytokine expression and chronic inflammation, which disrupts immune cell function and causes tissue damage [6].

Liver fibrosis results from chronic inflammation, causing hepatocyte loss and extracellular matrix (ECM) accumulation (primarily collagen and elastin) in the perisinusoidal space, with immune cells playing a crucial role [7]. Hepatic stellate cells (HSCs), the main ECM producers, are activated by profibrotic stimuli like ROS, transforming into myofibroblasts that produce collagen and inhibit matrix degradation [7] (Figure 1). Additionally, activated HSCs secrete pro-inflammatory cytokines and chemokines, activating and recruiting immune cells that further promote hepatic fibrogenesis [7] (Figure 1). In diabetes, profibrotic stimuli exacerbate inflammation, impair immune function, and activate HSCs, driving liver fibrosis [8] (Figure 1).

Immune cells, like monocytes/macrophages, neutrophils, invariant natural killer T (iNKT) cells, and T lymphocytes, influence the fibrotic microenvironment. Macrophages, including Kupffer cells (15% of hepatic cells), are key regulators of inflammation and fibrosis [9]. M1 (classically activated) macrophages promote inflammation, while M2 (alternatively activated) macrophages support anti-inflammatory effects and tissue repair [10]. Neutrophils facilitate inflammation resolution in tissues like the heart, skin, and joints [11,12,13], but can also contribute to liver fibrosis [14], with diabetes influencing their infiltration [15]. iNKT cells, a subset of NKT cells, interact with liver cells and may modulate hepatic metabolism [16], though their role in diabetes-related liver fibrosis remains uncertain. CD4+ and CD8+ T cells are vital in liver fibrosis, with CD8+ T cells causing liver damage [17] and CD4+ T cell subsets shaping the immune response [18].

These interactions highlight the role of immune cells, with population changes acting as markers and contributing to fibrosis progression. This systematic review explores immune responses in the liver, focusing on immune cell alterations in diabetes, cytokine profiles, and signaling pathways, and provides an overview of how these immune cells contribute to liver fibrosis in diabetes.

## 2. Materials and Methods

### 2.1. Search Protocol and Study Eligibility

The literature search used four databases: MEDLINE and Embase via OVID, PubMed, and Scopus. Only English full-text articles published up to June 2024 were included. The search strategy, including keywords and inclusion and exclusion criteria, was pre-defined by the authors (Table S1).

A keyword panel was designed with seven categories of terms and their variations (Table S1). Keywords related to diabetes, such as “diabetes mellitus”, “T1DM”, “T2DM”, and “diabetic” were searched alongside liver-related terms including “liver”, “hepatic”, “NASH”, “NAFLD”, “MAFLD”, “MASH”, “MASLD”, and “fatty liver”. Immune-related terms included “immune”, “immunity”, “immunophenotyping”, “adaptive”, and “innate immunity”, as well as specific immune cell types including “macrophage”, “T cell”, “B cell”, “neutrophil”, “monocyte”, “eosinophil”, “NK cell”, “dendritic cell”, “Kupffer cell”, “mast cell”, “leukocyte”, and “lymphocyte”. Additional terms covered species (“mice”, “mouse”, “murine”, “rat”, and “human”), fibrosis, and inflammation. Boolean operators combined synonyms with ‘OR’ and categories with ‘AND’.

**Figure 2:**
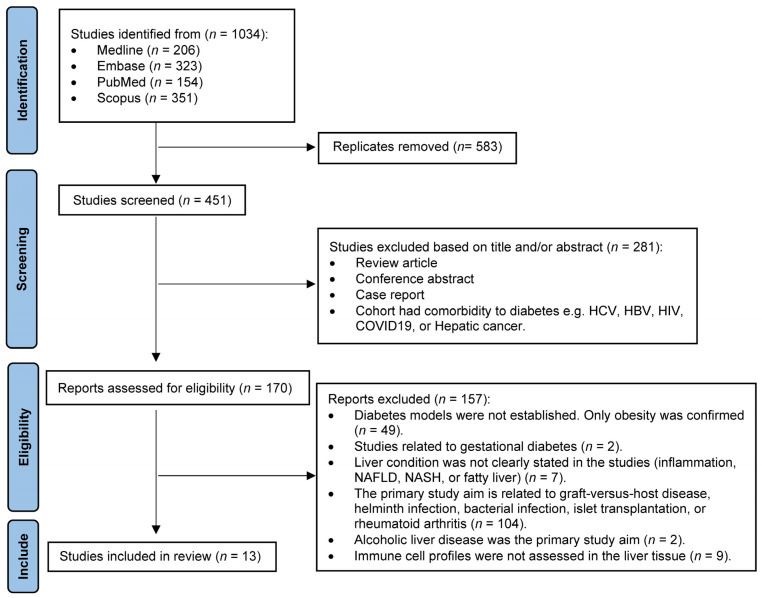
PRISMA flow diagram outlines the systematic literature search and selection process for studies profiling immune cells in the liver of people and animals with diabetes, encompassing both animal (mice and rats) and human research. From an initial 1034 studies identified, 583 duplicates were excluded. Screening of titles and abstracts resulted in the exclusion of 281 studies. A total of 170 full-text articles were assessed for eligibility, of which, based on exclusion criteria, 157 were excluded, whereas 13 met the predefined inclusion criteria and were included for comprehensive analysis.

Search results were exported to EndNote (v.21) for organization. Following the comprehensive search across all four databases, this systematic review was conducted in accordance with the Preferred Reporting Items for Systematic Reviews and Meta-Analyses (PRISMA) guidelines [19] (Figure 2). Duplicates were removed first; then, titles and abstracts were screened to exclude reviews, conference abstracts, case reports, and studies involving cohorts with comorbidities such as hepatitis C virus, hepatitis B virus, human immunodeficiency virus, coronavirus disease-19 infection, or hepatic cancer. Full texts of the remaining studies were screened further, excluding those where (i) diabetes was not confirmed or glucose/insulin tolerance tests were not conducted; (ii) subjects had gestational diabetes or alcoholic liver disease; (iii) the study did not focus on the liver (e.g., graft-versus-host disease, helminth or bacterial infections, islet transplantation, or rheumatoid arthritis); or (iv) immune cell profiles were not assessed in liver tissue. Two reviewers evaluated the remaining studies based on the inclusion criteria: (i) diabetes was confirmed; (ii) the study focused on liver tissue; (iii) immune cells were measured in the liver; and (iv) liver pathology was observed. The final number of studies included in the review is reported as ‘*n*’.

### 2.2. Data Extraction

Extracted data included the following variables: experimental and control groups, immune cell markers measured in liver tissue, cytokine expression levels in liver immune cells, signaling pathway activities identified in the liver, histopathological changes observed in liver tissue, and detection methods.

### 2.3. Risk of Bias Assessment

The Systematic Review Centre for Laboratory Animal Experimentation’s (SYRCLE’s) risk of bias tool was used to assess animal studies [20], covering ten criteria across seven bias types: selection, performance, detection, attrition, reporting, conflicts of interest, and other. The Newcastle-Ottawa Scale (NOS) was used to assess human case–control studies [21] across three domains: selection, comparability, and exposure.

## 3. Results

### 3.1. Search Results

The systematic search and selection process are summarized in Figure 2. The total number of papers found in four databases was 1034, using the keywords listed in Table S1. After removing duplicates (*n* = 583), 451 articles were screened by title and abstract. Applying the exclusion criteria, 281 articles were excluded, leaving 170 for full-text assessment. After further exclusions, 13 papers met the inclusion criteria and were included in the review (Figure 2).

Of these, 3 studies involved human participants, totaling 159 individuals aged 25 to 88 years [22,23,24]. Two studies investigated individuals with T2DM (*n* = 59, 5) [22,23], while one included both T1DM (*n* = 8) and T2DM (*n* = 16) individuals [24]. The NOS scores of these studies ranged from 8 to 9, reflecting good to high quality (Table S2). Ten studies used animal models, comprising 215 mice or rats [25,26,27,28,29,30,31,32,33,34]. Only one study utilized T1DM rats (*n* = 12) [32], while the remaining studies employed low-dose (50 mg/kg) STZ-induced diabetes (*n* = 6, 5, 10) [25,27,33] or T2DM animal models (*n* = 8, 60, 5, 10, 10, 4) [26,28,29,30,31,34]. SYRCLE’s assessment showed low to moderate bias risk (Table S3).

Investigated immune cells, including monocytes/macrophages (*n* = 8) [22,24,25,26,27,28,32,34], neutrophils (*n* = 3) [25,30,33], iNKT cells (*n* = 1) [29], and T cells (*n* = 5) [22,23,25,31,33], were evaluated at mRNA and protein levels.

### 3.2. Monocytes/Macrophages

Eight studies investigated monocytes/macrophages in the liver under diabetes, using nine markers to identify monocytes/macrophages in mice (CD11b, Ly6C, F4/80, CD11c, CD206, and CD86) and humans (CD14, CD16, and CD68) at protein or RNA levels (Table 1) [22,24,25,26,27,28,32,34]. The studies found increased monocytes/macrophages (CD11b+Ly6C^high^/F4/80+) in the livers of diabetic vs. non-diabetic mice [25,34]. In particular, the infiltrating macrophages (CD11b+F4/80^int^) were notably elevated (fold-change (FC) = 4.6, *p* < 0.01) in the livers of diabetes mice, along with a higher expression of interferon-ɣ (IFN-ɣ) (FC = 2, *p* < 0.01), tumor necrosis factor-α (TNF-α) (FC = 2.5, *p* < 0.05), and interleukin (IL)-1β (FC = 2, *p* < 0.01) [25] (Table 1). In contrast, liver-resident macrophages (CD11b+F4/80^high^) maintained similar populations in diabetic and non-diabetic mice [25] (Table 1).

Proinflammatory M1 macrophages (F4/80+CD11C−CD206− or CD86+) were significantly increased in diabetic vs. non-diabetic mice/rats (*p* < 0.05), and associated with liver abnormalities like steatosis, inflammation, necrosis, and fibrosis [26,27] (Table 1). Conversely, M2 macrophages (CD206+) showed a decrease in the livers of both T2DM and T1DM rats (*p* < 0.05), and were linked to liver pathology [27,32]. Notably, one study reported no significant changes in M2 macrophage populations in T2DM mice when using a different combination of M2 markers (F4/80+CD11C−CD206+) [26] (Table 1).

In human studies, non-classical CD14+CD16++ and intermediate CD14++CD16+ hepatic macrophages were more prevalent in diabetes with NASH or cirrhosis than diabetes alone [22] (Table 1). Furthermore, these macrophages exhibited higher IL-15 and IL-18 expressions in individuals with diabetes and NASH (1.7-fold and 2-fold, both *p* < 0.05, respectively) [24] (Table 1). Additionally, CD68+ macrophages were increased in both T1DM and T2DM individuals compared to healthy controls, with no significant difference between the two types of diabetes [24], consistent with findings from a study on diabetic rats [28] (Table 1).

One study demonstrated increased toll-like receptor 4 (TLR4)/myeloid differentiation primary response 88 (MyD88)/nuclear factor kappa-light-chain-enhancer of activated B cells (NF-κB) pathway activity in diabetic vs. non-diabetic rats, with higher TLR4 expression on hepatic macrophages (FC = 2.25, *p* < 0.05), contributing to steatosis, inflammation, hepatocyte ballooning and fibrosis in diabetes liver [28]. The AMP-activated protein kinase (AMPK)/mammalian target of rapamycin (mTOR) pathway was reported to be suppressed in diabetic mice, potentially reducing hepatic macrophage autophagy and increasing IL-17/IL-23 axis-related molecules’ expression, contributing to liver injury in T2DM with NAFLD [26] (Table 1).

### 3.3. Neutrophils

Three studies investigated neutrophils in the liver of mice with and without diabetes, using markers CD11b and Ly6G via flow cytometry [25,30,33]. The total neutrophil population was significantly higher in diabetic vs. non-diabetic mice (*p* < 0.05) (Table 1). Additionally, TNF-α and IL-1β levels in neutrophils increased 1.8- and 1.7-fold, respectively (*p* < 0.05) [25] (Table 1). These changes may contribute to steatosis, inflammation, and hepatocyte ballooning in diabetes [25,30,33].

### 3.4. iNKT Cells

A study investigated liver iNKT cells in mice using CD3 and NK1.1 via flow cytometry [29]. In diabetic mice, iNKT cells were reduced 1.8-fold vs. non-diabetic mice (*p* < 0.01) [29] (Table 1). Additionally, IL-4 expression in iNKT cells was lower in diabetic mice (*p* < 0.01) (Table 1), correlating with liver steatosis and inflammation [29].

### 3.5. T Cells

Five studies investigated T cells in the liver by flow cytometry using markers including CD4, CD8, CD45, CD69, CD28, CD57, programmed cell death protein-1 (PD-1), CD44, CD62L, CD25, and forkhead box P3 (Foxp3) [22,23,25,31,33] (Table 1).

In mouse studies, the proportion of CD4+ and CD8+ T cells in the liver was not affected by diabetes; however, the activated CD4+(CD69+) and CD8+(CD69+) T cell counts were higher in diabetes (FC = 2.5 and 2, respectively, both *p* < 0.05) vs. non-diabetes [25,33]. These activated T cells exhibited increased TNF-α expression (2- and 1.9-fold, respectively; both *p* < 0.01), associated with liver steatosis, inflammation, and hepatocyte ballooning [33] (Table 1). Additionally, regulatory T cells (Tregs) significantly increased in the liver of diabetes vs. non-diabetes (*p* < 0.01) [25], while CD4+CD44+CD62L− and CD8+CD44+CD62L− effector memory T cells were increased in diabetes with NASH compared to diabetes alone (both *p* < 0.01) [31] (Table 1). In contrast, CD4+(CD44−CD62L+) (*p* < 0.001) and CD8+(CD44−CD62L+) (*p* < 0.01) intrahepatic naïve T cells were lower in diabetes with NASH [31] (Table 1). Furthermore, IL-23 promotes the expansion and migration of CD4+ T cells in the liver of T2DM with NAFLD, leading to increased IL-17A expression and eventually liver injury [26] (Table 1).

In human studies, the senescent T cell proportion was more prevalent in individuals with T2DM and liver disease than those without liver disease [22,23]. CD4+CD28−CD57+ and CD8+CD28−CD57+ T cells increased 2.9- and 1.3-fold, respectively (both *p* < 0.05), in T2DM individuals with NASH or cirrhosis vs. those without liver disease [22]. Total senescent CD8+ T cells was higher in T2DM vs. healthy controls (*p* < 0.05) [23] (Table 1). PD-1 expression on CD4+ and CD8+ T cells also increased in T2DM (*p* < 0.05) [22], suggesting a higher exhausted T cell proportion in the liver (Table 1). IFN-γ and TNF-α expressions were elevated in senescent T cells (*p* < 0.05) [22] (Table 1).

## 4. Discussion

**Figure 3:**
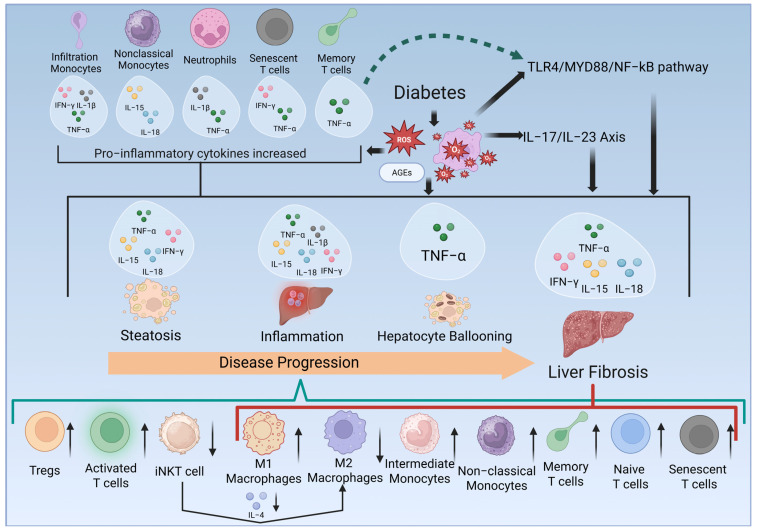
Proposed mechanism of immune dysregulation in diabetes-related liver fibrosis. This figure summarizes key immune cells, cytokines, and related pathways (TLR4/MYD88/NF-κB and IL-17/IL-23 axis) implicated in the progression of MAFLD/NAFLD, particularly MASH/NASH with fibrosis. Diabetes-induced ROS and AGEs drive steatosis, inflammation, and hepatocyte ballooning, promoting fibrosis which may be regulated by monocytes/macrophages and T cells [22,23,24,25,26,27,28,29,30,31,32,33,34]. Created with BioRender.com.

Diabetes and liver fibrosis are closely related, with metabolic dysfunction and chronic inflammation in diabetes likely driving liver fibrogenesis, where immune cells play a pivotal role [8]. This review clarifies the roles and regulations of liver immune cells in diabetes. Findings from human and animal studies underscore that diabetes, particularly T2DM, alters immune cell profiles, including monocytes/macrophages, neutrophils, iNKT and T cells, promoting chronic inflammation and liver disease progression. The updated proposed regulation of immune cells and their bioactivities in MAFLD/NAFLD, especially MASH/NASH with fibrosis, is illustrated in Figure 3. Table 2 summarizes liver pathology and the regulated immune cell subtypes, cytokines, and signaling pathways involved.

### 4.1. Innate Immune Cells

This review highlights a significant increase in infiltrating monocytes in diabetic livers, with hepatic macrophages shifting to a pro-inflammatory state, elevating IFN-γ, TNF-α, IL-1β, IL-15, and IL-18, promoting inflammation, and contributing to liver fibrosis [25] (Figure 3). Activated HSCs express chemokine ligand (CCL)-1, CCL2, and CCL25, recruiting monocytes [35], particularly Ly6C+ monocytes, from the circulation into the tissues [25,36]. Previous studies have shown that reducing liver-infiltrating monocytes inhibits fibrosis [37], implicating these cells as mediators of liver fibrogenesis [38]. In diabetes, HSCs can be activated by liver injury and systemic cytokines, like IL-17 and IL-20 [39,40], enhancing CCL2-mediated monocyte recruitment. Moreover, hyperglycemia can induce the proliferation and expansion of bone marrow myeloid progenitor cells, leading to increased circulating monocytes in diabetic mice [41]. This review supports the idea that increased monocyte infiltration is a key factor in the pathophysiology of diabetes-related liver disease.

The M1/M2 macrophage balance affects the liver microenvironment. Zhang et al. reported that activated HSC-derived CXCL10 promotes M1 polarization, while inhibiting CXCL10 can attenuate M1 polarization and reduce liver fibrosis [42]. In diabetes, this balance shifts toward pro-inflammatory M1 macrophages (Figure 3). An increased M1 macrophage population and decreased M2 phenotype in the blood of T2DM individuals [43] were consistent with the findings in the liver in this review [27,32]. However, it is noteworthy that M2 macrophage changes varied across studies. One possible explanation is the use of different animal models: Meng et al. utilized C57BL/6J mice [26], whereas others employed rats [27,32], which may exhibit distinct immune response under the same or similar stimuli [44].

Moreover, this review has identified a significant increase in CD14+CD16++ non-classical and CD14++CD16+ intermediate monocytes in the liver of T2DM individuals with NASH or cirrhosis [22], similar to Liaskou et al.’s study reporting an increase in these macrophage subsets in people with chronic inflammatory and fibrotic liver diseases, but without diabetes [45]. They suggested that these macrophages might arise either from the transmigration of CD14++CD16+ monocytes from the bloodstream or from local differentiation from classical CD14++CD16- monocytes within the liver [45].

Neutrophils were also significantly increased in the liver in diabetes [25,30,33] and contributed to hepatic inflammation by producing pro-inflammatory cytokines, activating hepatic macrophages, and recruiting other immune cells [46]. Neutrophil infiltration is common in various chronic liver diseases [14], driven by macrophage-secreted chemokines like CXCL1, CXCL2, and CXCL8 [47]. In diabetes, elevated free fatty acids, lipoproteins, and glucose contribute to a pro-inflammatory environment, which promotes neutrophil activation [15]. This inflammatory state and increased chemokine production in the liver enhance neutrophil recruitment and activation, supporting the emphasis in this review on their role in liver inflammation and fibrosis in diabetes.

The iNKT cells were significantly reduced in the liver in diabetes [29], however, their population in the liver was variously reported. A decrease in iNKT cells has been linked to impaired CD1d expression and lipid antigen presentation in the liver due to endothelial stress [48]. Additionally, Kupffer cells can induce the apoptosis and necrosis of overly-activated iNKT cells via IL-12 [49]. Conversely, Tajiri et al. observed increased iNKT cells in late stages of NAFLD, possibly from enhanced activation by Kupffer cells [50]. The effects of iNKT cells on the development of liver fibrosis remain controversial. On one hand, iNKT cells may promote liver fibrogenesis by activating HSCs through osteopathic expression, the Hedgehog signaling pathway [16], and by attracting neutrophils via IL-4 secretion [51]. However, on the other hand, iNKT cells can also kill early activated HSCs via an NKG2D-dependent mechanism, indicating a potential protective role against fibrosis [52]. The precise function of iNKT cells in diabetes-related fibrogenesis remains unclear and further research is required.

### 4.2. Adaptive Immune Cells

This review underscores the increased proportion of activated CD4+ and CD8+ T cells in the liver in diabetes [29]. In people with prediabetes, the concentration of cytokines that can activate T cells in the peripheral blood was increased, like TNF-α, IFN-γ, and IL-1β, compared to those with normoglycemia [23], potentially enhancing T cell activation, increasing the number of effector memory CD4+ and CD8+ T cells and reducing the percentage of naïve CD4+ and CD8+ T cells in the liver [31].

T cell senescence is also induced in T2DM and metabolic liver diseases. This review identified an increased proportion of senescent and exhausted CD4+ and CD8+ T cells in the liver of T2DM individuals with NASH or cirrhosis [22,23]. Normally, T cells undergo clonal expansion and differentiate into memory or effector cells throughout life [23]. However, chronic overstimulation impairs their function, leading to T cell senescence or ‘exhaustion’ [53]. In diabetes, people often exhibit elevated levels of T cell activators such as TNF-α and IFN-γ, along with low-grade chronic inflammation, both of which are known to induce T cell senescence [54]. These dysfunctional T cells contribute to insulin resistance and liver inflammation by secreting pro-inflammatory cytokines, activating HSCs, and promoting liver fibrosis [22].

In this review, it is evident that the population of Tregs is significantly increased in the liver in diabetes [25]. Tregs are essential for maintaining peripheral immune tolerance and regulating immune responses [55]. HSCs may selectively promote CD4+CD25+Foxp3+ Tregs expansion via IL-2 [56], which can interpret the MMP/TIMP balance, influence Kupffer cell activity, and drive ECM remodeling and fibrogenesis, possibly through the activation of HSCs by transforming growth factor-β (TGF-β) [57,58]. However, Tregs can also inhibit fibrosis through IL-10, which can reduce collagen I synthesis [59], limit Kupffer cell activation [60], decrease IL-17 via Th17 cells, and reduce HSC activation and fibrosis [61]. This dual role highlights the complexity of Tregs in fibrosis, balancing tissue repair and inflammation.

Supporting this protective role, Treg depletion increased IFN-γ and TNF-α expression [25], promoting monocyte and neutrophil infiltration and exacerbating liver inflammation, suggesting that Tregs may help prevent the progression of liver fibrosis in diabetes. Additionally, Treg expansion also occurs in the thymus and peripheral tissues of diabetic mice [62], likely as a compensatory response to the chronic inflammation and immune dysregulation characteristic of diabetes. Overall, Tregs play a crucial role in liver fibrosis in diabetes.

### 4.3. Related Cytokines and Signalling Pathways

Cytokine expression and signaling pathway activation drive liver fibrosis in diabetes. This review identified key changes in IFN-ɣ [23,25] and TNF-α [23,25,27,28,34], produced by monocytes, CD4+ and CD8+ T cells, and neutrophils. IL-1β [25,28,33,34], IL-4 [29], IL-15 [22,29], and IL-18 [22] also exhibit significant changes. Additionally, the TLR4/MyD88/NF-κB pathway [28] and IL-17/IL-23 axis [26] were identified as crucial players in liver fibrogenesis in diabetic mice.

TNF-α promotes fibrogenesis by activating HSCs and Kupffer cells, which contribute to hepatocyte death and drive ECM deposition, a key process in fibrosis development [63]. TNF-α triggers caspase-mediated cell death and the NF-κB pathway, which regulates cell survival and the expression of pro-inflammatory cytokines [64] (Figure 3). These combined actions of TNF-α promote a cycle of inflammation and likely then magnify fibrosis within the liver [63]. In diabetes, an elevated TNF-α level amplifies inflammatory responses, creating a sustained pro-inflammatory microenvironment that exacerbates chronic liver injury and accelerates fibrosis progression. Additionally, IFN-γ, mainly from activated T cells, promotes hepatocyte damage by driving pro-inflammatory responses, particularly via CD8+ T cells, which are cytotoxic and have been implicated in liver damage [65]. Together, TNF-α and IFN-γ drive persistent inflammation and HSC activation, accelerating fibrosis.

IL-1β, IL-15, and IL-18 also contribute to liver fibrosis. IL-1β, primarily secreted by neutrophils and monocytes/macrophages, worsens inflammation, recruits immune cells, and activates HSCs [65], fueling a self-perpetuating cycle of liver injury and fibrosis. IL-15 enhances the activation of hepatic macrophages and mediates the functions of CD8+ T cells, contributing to fibrogenesis in the liver [66,67]. Also, IL-18 affects the functions of T cells and activates HSCs to promote the fibrotic process in the liver [68].

IL-4, produced by iNKT cells, is critical mediator of the anti-inflammatory immune response. As one Th2 cytokine, it plays a key role in suppressing inflammation and promoting the polarization of macrophages toward the M2 phenotype, which has anti-inflammatory properties [69]. However, in diabetes, the expression of IL-4 is significantly reduced, which may hinder M2 polarization and prolong chronic inflammation, ultimately exacerbating liver fibrosis [29]. The hepatic immune cell regulation implicated in liver fibrosis in diabetes is shown schematically in Figure 3.

Beyond cytokine dysregulation, key signaling pathways were identified in this review, notably the TLR4/MyD88/NF-κB pathway and the IL-17/IL-23 axis [28]. The TLR4/MyD88/NF-κB pathway is a central mediator of innate immune responses and is often activated by pathogen-associated molecular patterns and damage-associated molecular patterns in the liver [70]. The activation of this pathway in diabetes leads to the production of pro-inflammatory cytokines and further recruitment of immune cells, thus sustaining chronic inflammation and promoting fibrosis [71]. This further suggests that monocytes/macrophages, as primary players in this pathway [70], likely act as key initiators of the inflammatory cascade that accelerates liver fibrogenesis in diabetic conditions (per Figure 3).

The IL-17/IL-23 axis also plays a crucial role as noted in this review [26]. IL-17, secreted by Th17 cells, activates HSCs and promotes collagen production, driving fibrotic scarring [72]. The increased activation of this axis in diabetes, also as shown in Figure 3, suggests a heightened role for adaptive immunity, where CD4+ T cells, particularly the Th17 subset, exacerbates the fibrotic response by sustaining inflammation and promoting the activation of HSCs.

### 4.4. Limitations

This review is limited by significant heterogeneity across the studies, including variations in methodologies for assessing immune cells and inflammatory markers (e.g., flow cytometry and immunohistochemistry), marker selection, sample types, disease model, and liver pathology severity. Some studies focused on specific immune cells, while others used broader categories, complicating direct comparisons. Assay sensitivity and specificity for inflammatory markers also varied, contributing to inconsistent findings. As shown in Figure 3, current data do not clarify which immune cells are most linked to severe liver fibrosis. Another limitation is the lack of diabetes stratification, with few studies distinguishing long-standing, well-controlled, and poorly controlled diabetes. This limits understanding of immune dysregulation and its link to liver pathology.

A further limitation is the limited characterization of hepatic immune cell differences between T1DM and T2DM. Although these conditions have distinct pathogenesis, with the autoimmune destruction of β-cells in T1DM [73] and chronic inflammation driven by metabolic dysfunction in T2DM [74], comparative data on liver immune profiles remain sparse. This review includes only one human and one animal study on T1DM, identifying M2 and CD68+ macrophages [24,32], with findings consistent with those observed in T2DM. Further studies are needed to clarify liver-specific immune alterations in both types of diabetes.

This review sought to provide comprehensive mechanistic insights, but no studies examined immune cell localization in the liver, crucial for understanding their roles in hepatic fibrogenesis. Additionally, although we focused on immune cell profiles in the liver in diabetes in this review, only cytokines directly linked to these immune cells were included, in line with the inclusion criteria. As a result, several other cytokines, such as TGF-β, IL-22, IL-10, and IL-6, which are known to play important roles in liver fibrosis [14], were not covered, as they were not addressed in the eligible studies. Future research could explore these cytokines in the liver to provide a more comprehensive understanding of their interactions with immune cells in liver fibrosis.

## 5. Conclusions

In conclusion, this review identified an increase in monocytes/macrophages, neutrophils, and CD4+ and CD8+ T cells and a reduction in iNKT cells in the liver of individuals and animals with diabetes. These shifts were associated with liver pathology, suggesting a role for immune cells in liver fibrosis progression in diabetes. Given the diversity of immune cells, cytokine functions, and unclear links to liver pathologies, further research is needed to understand how immune responses and hepatic cells drive liver fibrosis in diabetes and whether targeting specific immune cells can prevent or normalize MASH/NASH and liver fibrosis.

## Figures and Tables

**Table 1:** Summary of immune cell population changes, marker expression, related cytokines’ expression, signaling pathways, and pathological correlations in the liver with diabetes [22,23,24,25,26,27,28,29,30,31,32,33,34].

Cell Types	Marker Detected	Direction of Change	Fold-Change	*p* Value	Altered Cytokines	Activated Pathways	Mice/Rats Models */Human Studies	Reported Liver Pathology
Monocytes/Macrophages	CD11b+Ly6C^high^	Increased	2.8 [25]	<0.01		IL-17/IL-23 Axis [26] and TLR4/MyD88/NF-κB pathway [28]	Normal chow-fed, STZ-induced diabetic mice vs. normal chow-fed mice	Inflammation
	CD11b+F4/80^int^	Increased	4.6 [25]	<0.01	↑IFN-γ [25]			
					↑TNF-α [25]			
					↑IL-1β [25]			
	CD11b+F4/80^high^	No significant change	N/A [25]	>0.05				
	F4/80+CD11c+CD206-	Increased	6.5 [26]	<0.01			High-fat-diet-fed, STZ-induced T2DM mice vs. normal-chow-diet-fed mice	Steatosis and inflammation
	F4/80+CD11c-CD206+	No significant change	N/A [26]	>0.05				
	CD86+	Increased	4.5 [26]	<0.01				
			2.2 [27]	<0.05			High-fat-diet-fed, STZ-induced diabetic Wistar rats vs. normal-chow-diet-fed Wistar rats	Inflammation and necrosis and fibrosis
	CD206+	Decreased	3.9 [27]	<0.05				
			2 [32]	<0.01			Diabetes-prone Bio Breeding rats (T1DM) vs. Bio Breeding rats	Steatosis and inflammation
	CD14++ CD16+^int^	Increased	NASH: 2.8 [22]	<0.001			Humans with T2DM and with or without NASH or liver cirrhosis	Steatosis and inflammation and fibrosis
			Cirrhosis:2.3 [22]	<0.001				
	CD14+CD16++	Increased	NAHS: 1.3 [22]	<0.05	↑IL-18 and ↑IL-15 [22]			
			Cirrhosis:2.5 [22]	<0.01				
	CD68+	Increased	2 [24]	<0.001			Humans with or without T1DM/T2DM	Steatosis observed in T2DM
			N/A [28]	N/A			High-fat-diet-fed, STZ-induced T2DM rats vs. normal-chow-fed rats	Steatosis and inflammation and hepatocyte ballooning and fibrosis
	F4/80+	Increased	1.3 [34]	<0.005			High-fat-diet-fed T2DM mice vs. normal-chow-diet-fed mice	Inflammation
Neutrophils	CD11b+Ly6G+	Increased	1.5 [25]	<0.05	↑TNF-α [25]		Normal chow-fed, STZ-induced diabetic mice vs. normal chow-fed mice	
					↑IL-1β [25]			
			1.5 [30]	<0.05				
			6 [33]	<0.001			High-fat-diet-fed T2DM mice vs. normal-chow-diet-fed mice	Steatosis and inflammation and hepatocyte ballooning
iNKT cells	CD3+NK1.1+	Decreased	1.8 [29]	<0.01	↓IL-4 [29]		Normal chow-fed, KK-Ay diabetic mice vs. glycine-containing diet-fed KK-Ay mice	Steatosis and inflammation
T cells	CD45+CD4+	No significant change	N/A [25]	>0.05		IL-17/IL-23 Axis [26]	Normal chow-fed, STZ-induced diabetic mice vs. normal chow-fed mice	Inflammation
	CD45+CD8+	No significant change	N/A [25]	>0.05				
	CD4+CD69+	Increased	2.5 [33]	<0.05				Steatosis and inflammation and hepatocyte ballooning
	CD8+CD69+	Increased	2 [33]	<0.05				
	CD4+CD28-CD57+	Increased	2.9 [22]	<0.05	↑TNF-α [22]		Individuals with T2DM and with or without NASH or liver cirrhosis	Steatosis and inflammation and fibrosis
	CD8+CD28-CD57+	Increased	1.3 [22]	<0.05	↑IFN-γ [22]			
					↑TNF-α [22]			
			1.4 [23]	<0.05			Individuals with or without T2DM	Inflammation
	CD4+PD-1+	Increased	NASH: 1.3 [22]	<0.01			Individuals with T2DM and with or without NASH or liver cirrhosis	Steatosis and inflammation and fibrosis
			Cirrhosis:1.7 [22]	<0.01				
	CD8+PD-1+	Increased	NASH: 1.4 [22]	<0.05				
			Cirrhosis:2 [22]	<0.01				
	CD4+CD44+CD62L-	Increased	1.4 [31]	<0.01	↑TNF-α [31]		High-fat-diet-fed diabetic mice within specific-pathogen-free (T2DM) environment vs. exposed to open environment	Steatosis and inflammation and hepatocyte ballooning and fibrosis
	CD8+CD44+CD62L-	Increased	1.5 [31]	<0.001	↑TNF-α [31]			
	CD4+CD44-CD62L+	Decreased	8.5 [31]	<0.001				
	CD8+CD44-CD62L+	Decreased	3.4 [31]	<0.01				
	CD25+Foxp3+	Increased	4.5 [25]	<0.01			Normal chow-fed, STZ-induced diabetic mice vs. normal chow-fed mice	Inflammation

* Experimental group vs. control group. ↑ indicates significant increase, ↓ indicates significant decrease in cytokine levels.

**Table 2:** Summary of findings of liver pathology, along with immune cell type, cytokines, and signaling pathway dysregulation in the liver [22,23,24,25,26,27,28,29,30,31,32,33,34].

	Liver Pathology	Steatosis	Inflammation	Hepatocyte Ballooning	Necrosis	Fibrosis
Immune Factors						
Immune cells increased		M1 macrophages [26]	Infiltration monocytes [25]	Neutrophils [34]	M1 macrophages [27]	M1 macrophages [27]
			M1 macrophages [26,27]			
		Intermediate monocytes [22]	Intermediate monocytes [22]			Intermediate monocytes [22]
		Non-classical monocytes [22]	Non-classical monocytes [22]			Non-classical monocytes [22]
		Neutrophils [34]	Neutrophils [25,33,34]			
		Activated CD4+/CD8+ T cells [33]	Activated CD4+/CD8+ T cells [33]	Activated CD4+/CD8+ T cells [33]		
		Senescent CD4+/CD8+ T cells [22]	Senescent CD4+/CD8+ T cells [22,23]			Senescent CD4+/CD8+ T cells [22]
		PD-1+CD4+/CD8+ T cells [22]	PD-1+CD4+/CD8+ T cells [22]	Memory CD4+/CD8+ T cells [31]		PD-1+CD4+/CD8+ T cells [22]
		Memory CD4+/CD8+ T cells [31]	Memory CD4+/CD8+ T cells [31]			Memory CD4+/CD8+ T cells [31]
			T regulatory cells [25]			
Immune cells decreased		M2 macrophages [32]	M2 macrophages [27,32]	Naïve CD4+/CD8+ T cells [31]	M2 macrophages [27]	M2 macrophages [27] Naïve CD4+/CD8+ T cells [31]
		iNKT cells [29]	iNKT cells [29]			
		Naïve CD4+/CD8+ T cells [31]	Naïve CD4+/CD8+ T cells [31]			
Cytokines upregulated		IFN-γ [22] TNF-α [22,31] IL-18 [22] IL-15 [22]	IFN-γ [22,25] TNF-α [22,25,31] IL-18 [22] IL-15 [22] IL-1β [25]	TNF-α [31]		IFN-γ [22] TNF-α [22,31] IL-18 [22] IL-15 [22]
Cytokines downregulated		IL-4 [29]	IL-4 [29]			
Signaling pathways activated		IL-17/IL-23 axis [26]	IL-17/IL-23 axis [26]	TLR4/MyD88 pathways [28]		TLR4/MyD88 pathways [28]
		TLR4/MyD88 pathways [28]	TLR4/MyD88 pathways [28]			

## Supplementary Materials

The supporting information can be downloaded at https://www.mdpi.com/article/10.3390/ijms26094027/s1.

## Abbreviations

The following abbreviations are used in this manuscript:

AP-1	activator protein-1
AGEs	advanced glycation end products
AMPK	AMP-activated protein kinase
ECM	extracellular matrix
Foxp3	forkhead box P3
HSCs	hepatic stellate cells
IFN-ɣ	interferon-ɣ
IL	interleukin
iNKT	invariant natural killer T
mTOR	mammalian target of rapamycin
MMPs	matrix metalloproteinases
MAFLD	metabolic dysfunction-associated fatty liver disease
MASH	metabolic dysfunction-associated steatohepatitis
MASLD	metabolic dysfunction-associated steatotic liver disease
MyD88	myeloid differentiation primary response 88
NOS	Newcastle-Ottawa Scale
NAFLD	non-alcoholic fatty liver disease
NASH	non-alcoholic steatohepatitis
NF-κB	nuclear factor-kappa B
PRISMA	Preferred Reporting Items for Systematic Reviews and Meta-Analyses
PD-1	programmed cell death protein-1
ROS	reactive oxygen species
Tregs	regulatory T cells
SYRCLE	Systematic Review Centre for Laboratory Animal Experimentation
TIMPs	tissue inhibitors of metalloproteinases
TLR4	toll-like receptor 4
TGF-β	transforming growth factor-β
TNF-α	tumor necrosis factor-α
T1DM	type 1 diabetes mellitus
T2DM	type 2 diabetes mellitus
