# Supplementary material for: Alterations in Immune Cell Profiles in the Liver in Diabetes Mellitus: A Systematic Review
Source: Int J Mol Sci. 2025 Apr 24;26(9):4027. doi: 10.3390/ijms26094027 (PMC12071842; doi:10.3390/ijms26094027)
Supplement: Supplementary file 1 [file ijms-26-04027-s001.zip › ijms-3556626-supplementary.pdf]

Supplementary Table S1. Keywords and their variants for literature search in Embase, Medline, PubMed and Scopus, as well as predefined inclusion and exclusion criteria.

Diabetes	Immune	Liver	Mice	Human	Fibrosis	Immune cells	Inclusion	Exclusion
Diabetes mellitus	Immuno	NASH	Mouse		Inflammation	Macrophage	Including all the key words	HIV, HBV, HCV, Covid19 infection related
T1DM	immunophenotyping	Hepatic	Murine		Inflamm*	Monocyte	Must be specific on diabetes or not (ITT and GTT tests can be accepted)	Hepatic cancer related
T2DM	Immune response	NAFLD			Inflammatory	Kupffer cell	Must be specific on immune cells (whether focused on cell markers or other genes that can be used to detect cells)	Not specific on whether the sample have diabetes or not
diabetic	Immunity	Fatty liver				NK cell	Must within liver tissue	Related to gestational diabetes
Diabet*	Adaptive immunity	MAFLD				eosinophil	Liver tissue should show at least inflammation at some grade	Liver condition was not clearly stated
Type 1 DM	Innate immunity	MASH				Dendritic cell	For studies that have treatments, as long as the non-treatment group meet the criteria, it can be included	Alcoholic liver disease was studied
Type 2 DM		MASLD				Neutrophil		Other organ related studies
Pre-diabetes						Mast cell		Immune cells profiles were not assessed in the liver tissue
						Leukocyte		
						T cell		
						B cell		
						Lymphocyte		

Supplementary Table S2. NOS study quality assessment for Case-Control studies included in the review.

Study	Study Design	Selection*	Comparability¢	Exposure¶	Total Score§
**Sim et al., 2023**	Case Control	4	2	3	9
**Yi et al., 2019**	Case Control	4	2	3	9
**Korn et al., 2023**	Case Control	3	1	2	8

*Maximum 4 points awarded for selection with four prospective: case definition, representativeness of the cases, selection of controls, and definition of controls.

¢Maximum 2 points awarded for comparability of cases and controls based on design or analysis.

¶Maximum 3 points awarded for exposure with three prospective: ascertainment of exposure, same method of ascertainment for cases and controls, and non-response rate of participants.

§A maximum of 9 points could be awarded.

Supplementary Table S3. Risk of bias assessment for each animal studies included in the review. Selection, performance, detection, attrition, reporting, conflicts of interest, and other biases were included.

Study (Author,	Sequence	Baseline	Allocation	Random Housing	Blinding of Caregivers	Blinding of Outcome	Incomplete Outcome Data	Selective Outcome	Other Potential	Funding of Conflicts of	Overall RoB
Lee et al., 2016	Low	Low	Low	Low	High	High	Low	Low	Unclear	Low	Low
Meng et al., 2021	Low	Low	Low	Low	High	High	Unclear	Unclear	Unclear	Low	Moderate
Sheikh et al., 2023	Low	Unclear	Low	Low	High	High	Low	Low	Unclear	Low	Moderate
Han et al., 2016	Low	Unclear	Low	Low	High	High	Unclear	Unclear	Unclear	Low	Moderate
Takashima et al., 2015	Low	Low	Low	Low	High	High	Low	Low	Unclear	Low	Moderate
Liu et al., 2018	Low	Unclear	Low	Low	High	High	High	Unclear	Unclear	Low	Low
Kind, et al., 2019	Low	Unclear	Low	High	High	High	Unclear	Unclear	Unclear	Low	Moderate
Serrano et al., 2019	Low	Low	Low	Low	High	High	Unclear	Unclear	Unclear	Low	Moderate
Chen et al., 2021	Low	Low	Low	Low	High	High	Unclear	Unclear	Unclear	Low	Low
Xu et al., 2013	Low	Low	Low	Low	High	High	Unclear	Unclear	Unclear	Low	Low
